# Hcp1-loaded staphylococcal membrane vesicle vaccine protects against acute melioidosis

**DOI:** 10.3389/fimmu.2022.1089225

**Published:** 2022-12-23

**Authors:** Keting Zhu, Gang Li, Jia Li, Mingxia Zheng, Xiaohui Peng, Yifan Rao, Ming Li, Renjie Zhou, Xiancai Rao

**Affiliations:** ^1^ Department of Emergency Medicine, Xinqiao Hospital, Army Medical University, Chongqing, China; ^2^ Department of Microbiology, College of Basic Medical Sciences, Army Medical University, Chongqing, China

**Keywords:** *Burkholderia pseudomallei*, vaccine, membrane vesicles, Hcp1, melioidosis, protection

## Abstract

*Burkholderia pseudomallei* is the causal agent of melioidosis, a deadly tropical infectious disease that lacks a vaccine. On the basis of the attenuated *Staphylococcus aureus* RN4220-Δ*agr* (RN), we engineered the RN4220-Δ*agr*/*pdhB-hcp1* strain (RN-Hcp1) to generate *B. pseudomallei* hemolysin-coregulated protein 1 (Hcp1)-loaded membrane vesicles (^hcp1^MVs). The immunization of BALB/c mice with ^hcp1^MVs mixed with adjuvant by a three-dose regimen increased the serum specific IgG production. The serum levels of inflammatory factors, including TNF-α and IL-6, in ^hcp1^MV-vaccinated mice were comparable with those in PBS-challenged mice. The partial adjuvant effect of staphylococcal MVs was observed with the elevation of specific antibody titer in ^hcp1^MV-vaccinated mice relative to those that received the recombinant Hcp1 protein (rHcp1) or MVs derived from RN strain (^Δagr^MVs). The ^hcp1^MVs/adjuvant vaccine protected 70% of mice from lethal *B. pseudomallei* challenge. Immunization with ^hcp1^MVs only protected 60% of mice, whereas vaccination with rHcp1 or ^Δagr^MVs conferred no protection. Moreover, mice that received ^hcp1^MVs/adjuvant and ^hcp1^MVs immunization had low serum TNF-α and IL-6 levels and no inflammatory infiltration in comparison with other groups. In addition, all surviving mice in ^hcp1^MVs/adjuvant and ^hcp1^MVs groups exhibited no culturable bacteria in their lungs, livers, and spleens five days postinfection. Overall, our data highlighted a new strategy for developing *B. pseudomallei* vaccine and showed that Hcp1-incorporated staphylococcal MV is a promising candidate for the prevention of acute melioidosis.

## Introduction

*Burkholderia pseudomallei* is a dangerous pathogen that causes melioidosis, a tropical infectious disease with clinical manifestation varying from local abscess to systemic sepsis (1). Human melioidosis was first described in 1911 by Dr. Alfred Whitmore in Yangon, Myanmar, and this disease has been neglected until 1985 when the Infectious Disease Association of Thailand has enabled people to recognize melioidosis as a remarkable public health problem (2, 3). The worldwide distribution of *B. pseudomallei* especially in tropical areas such as South America and the Caribbean, Southeast Asia, Northern Australia, and the Indian subcontinent, results in an estimate of 165,000 human melioidosis cases per year and a high mortality of 53.9% (2–5). *B. pseudomallei* can colonize and invade multiple organs of the body, including brain, lung, liver, kidney, and skin, and the extensive tissue tropism of *B. pseudomallei* contributes to the development of melioidosis (6, 7) and complicates the diagnosis due to the variable clinical presentations of the disease (2). Moreover, *B. pseudomallei* is intrinsically resistant to a variety of antibiotics (2, 8), and insufficient antibacterial treatment can lead to a fatality rate of more than 70% (9, 10). In addition, *B. pseudomallei* has been recognized as a category B biological agent by the US Centers for Disease Control and Prevention (11). The high prevalence and potential biological threat of *B. pseudomallei* calls for effective vaccines. However, commercial vaccines against melioidosis are still unavailable.

Substantial effort has been given to the development of melioidosis vaccines in recent years. Preclinical studies showed that inactivated whole-cell (such as paraformaldehyde-killed *B. pseudomallei* A2 (12)) and live attenuated (like the auxotrophic mutant Δ*ilvI* (13)) vaccines can protect 20%–80% of vaccinated mice from lethal bacterial challenge. However, sterilizing immunity is not achieved (8). By contrast, subunit vaccines with one or several antigens are considered safe and protective. Therefore, the selection and assessment of potential antigen candidates from *B. pseudomallei* have attracted the attention of the research community. Many antigens, such as the lipoprotein export system component LolC (14), outer membrane protein OmpW (15), heat shock protein GroEL (16), trimeric autotransporter adhesin PSL2063 (17), type III secreted protein BopA (18), and the type VI secretion system (T6SS)-associated hemolysin-coregulated proteins (Hcp; including Hcp1, Hcp2, Hcp3, and Hcp6) (19), have been identified as good vaccine candidates for further investigation. However, these subunit vaccine candidates have not advanced to clinical trials due to lack of suitable delivery systems.

Bacterial membrane vesicles (MVs) are nanoscale structures naturally secreted by Gram-positive bacteria and Gram-negative ones (also termed outer membrane vesicles, OMVs) during their growth (20). MVs can incorporate bacterial proteins, which may effectively stimulate specific humoral and cellular immune responses, and grant remarkable protection against subsequent bacterial infections (21). The innate characteristics of bacterial MVs support vaccine candidates and provide excellent vaccine delivery systems (22, 23). Examples of the successful bacterial MV vaccines are two licensed meningococcal serogroup B (MenB) MV vaccines designated as MenB-4C and MenB-FHbp, which can protect against infections caused by all 14 pathogenic meningococcal strains tested (24). A safe staphylococcal platform has been constructed previously by deleting the whole *agr* locus in the genome of *Staphylococcus aureus* strain RN4220, and at least four dominant components in the RN4220-Δ*agr* mutant are capable of delivering the antigens of dengue virus to MVs, which can induce specific antibodies against all four serotypes of the dengue virus (25).

As one of the subunit vaccine candidates against melioidosis, Hcp1 is a structural protein forming the secretion tube of T6SS and an important effector involving in *B. pseudomallei* pathogenesis (26). Anti-Hcp1 specific antibodies, including IgM and IgG, exist in the sera of patients with melioidosis, indicating the development of humoral response against Hcp1 (26, 27). In the present study, the *hcp1* gene is genetically in-frame fused to the gene encoding pyruvate dehydrogenase E1 component subunit beta (PdhB), one of the aforementioned major vesicular components in *S. aureus* RN4220-Δ*agr*. The immunization of BALB/c mice with Hcp1-loaded MVs derived from RN4220-Δ*agr/pdhB-hcp1* (^hcp1^MVs) induces a high titer of specific antibodies and protects about 70% of vaccinated mice from acute intraperitoneal challenge with lethal *B. pseudomallei*, highlighting a technical advancement in developing melioidosis vaccines.

## Materials and methods

### Plasmids, bacterial strains, and their growth conditions

The plasmids and bacterial strains used in this study were listed in **Supplementary Table 1**. *S. aureus* strains were cultured from a freezer stock and streaked on tryptic soy broth (TSB; Oxoid, UK) agar for 16 h at 37°C. The colonies of bacteria were inoculated into 2 mL of TSB and incubated for 16 h at 37°C with agitation. *Escherichia coli* strains were grown in Luria Broth (LB) medium or plated on LB agar for 16 h at 37°C. *B. pseudomallei* strains were cultured from a freezer stock and spread on LB agar supplemented with 200 μM FeSO_4_·7H_2_O for 48 h at 37°C. Bacterial colonies were then inoculated into 2 mL of LB broth and cultured at 37°C for 16 h with agitation. When required, cultures were supplemented with 100 μg/mL of kanamycin (Kan) or 50 μg/mL of ampicillin (Amp) for *E. coli* and 10 μg/mL of chloramphenicol (Cm) for *S. aureus* harboring recombinant plasmids.

The plasmid pBT2 and *S. aureus* RN4220 were kindly provided by Prof. Baolin Sun (University of Science and Technology of China). *B. pseudomallei* strain BPC006 was isolated from the clinical specimen of a patient hospitalized in Hainan People’s Hospital (28), and kindly provided by Prof. Xu-hu Mao (Army Medical University).

### Construction of *S. aureus* strain producing Hcp1-loaded MVs

To construct *S. aureus* RN4220-Δ*agr/pdhB-hcp1*, the *hcp1* gene was amplified from *B. pseudomallei* BPC006 genomic DNA using primer pair hcp1-F/R (**Supplementary Table 2**). For homologous recombination, the left region (Up-pdhB) and right region (Down-pdhB) across the stop codon of the *pdhB* gene of *S. aureus* RN4220-Δ*agr* were amplified from the genomic DNA using primers uppdhB-F/R and downpdhB-F/R (**Supplementary Table 2**), respectively. A fusion fragment was generated by overlap PCR using the *hcp1* gene fragment, Up-pdhB, and Down-pdhB as templates. The fusion DNA was digested with *Bam*H I/*Sac* I restriction enzymes and ligated into the temperature-sensitive shuttle vector pBT2 *via* Gibson assembly master mix (NEB, USA). The resultant vector pBT2-hcp1 was firstly transformed into *E. coli* DH5α and subsequently electroporated into *S. aureus* strain RN4220-Δ*agr.* The seamless *hcp1* insertion mutant designated as RN4220-Δ*agr/pdhB-hcp1* (RN-Hcp1) was screened as previously described (29), and confirmed through PCR amplification and DNA sequencing.

### Preparation of bacterial MVs

Bacterial MVs were prepared from *S. aureus* culture supernatants as described previously (25). Briefly, overnight culture of *S. aureus* strains of interest was inoculated into 1 L TSB (1:100), and incubated for 20 h at 37°C with shaking (200 rpm). Culture supernatants were collected by centrifugation at 5,000 × *g* at 4°C for 30 min to remove bacterial cells, and then filtered through 0.45 μm Millex syringe filters (Beyotime) to remove any remaining cell debris. The filtrate was concentrated about 60 times with an ultrafiltration system with an interception molecular weight of 100 kDa (Shenchen, China). Culture supernatants were then centrifuged at 200,000 × *g* for 3 h at 4°C, and the MV pellets were resuspended in phosphate-buffered saline (PBS, pH 7.2) after two washes with PBS. The protein concentration of MV samples was determined using an enhanced BCA Protein Assay Kit according to the instruction provided by the Company (Beyotime). MVs were stored as aliquots at -80°C.

### Transmission electron microscope (TEM) observation

The purified Hcp1-loaded MVs were added to 230-mesh formvar/carbon-coated copper grids (Zhongjingkeji Tech., China), and negatively stained with 2% (m/v) uranylacetate for 30 sec. For immunogold labeling, Hcp1-loaded MVs was 1:20 diluted in NTE buffer (10 mM Tris-Cl, pH 7.0, 100 mM NaCl) and adsorbed onto 230-mesh formvar/carbon-coated nickel grids. After 5 min wash with TBS buffer (50 mM Tris-HCl, 150 mM NaCl, pH 7.5), the specimen was blocked with 3% (m/v) bovine serum albumin (BSA) in TBS for 45 min. Mouse-anti-Hcp1 antibody was 1:500 diluted in 1% (m/v) BSA/TBS and applied to the nickel grids for 1.0 h at room temperature. After washed three times with TBS, gold-conjugated goat-anti-mouse IgG (Sigma, USA) was added and incubated for 1.0 h at room temperature. Next, the grids were washed twice with TBS, once with water and followed by negatively stained with 2% (m/v) uranylacetate for 15 sec. Electron micrographs were recorded with a JEM1011 microscope (JEOL, Japan) at 100 kV acceleration voltage.

### Expression and purification of recombinant Hcp1

Recombinant Hcp1 (rHcp1) was expressed and purified as described previously with minor modifications (25). Briefly, the *hcp1* gene was amplified from genomic DNA of *B. pseudomallei* BPC006 using primer pair pET28a-F/R (**Supplementary Table 2**), and cloned into the expression vector pET28a to generate pET28a-*hcp1*. *E. coli* strain BL21(DE3) harbouring the resulting plasmid pET28a-*hcp1* was grown in 1.0 L fresh LB broth at 37°C to an OD600 of 0.5 and induced with 0.5 mM IPTG at 37°C for 6 h. For protein purification, bacterial cells were harvested by centrifugation at 5,000 × g at 4°C for 30 min and lysed by sonication in buffer A (50 mM Tris-HCl and 200 mM NaCl, pH 8.0). After centrifugation at 16,000 × g at 4°C for 30 min to remove cell debris, the supernatant was collected and centrifuged again at 10,000 × g for 30 min, filtered through a 0.22 mm filter unit (Beyotime), and applied to an Ni-NTA resin column (GE, USA) pre-equilibrated with buffer A. The column was washed with buffer A, followed by buffer A mixed with a linear gradient of 5%–100% (v/v) buffer B (buffer A containing 500 mM imidazole) to remove the nonspecifically bound proteins and elute the target proteins. Protein purity was analyzed by sodium dodecyl sulfate polyacrylamide gel electrophoresis (SDS-PAGE), and the major fractions containing target proteins were collected and dialyzed five times against buffer A to remove imidazole. The concentration of rHcp1 proteins was determined using a BCA protein assay kit (Pierce). Aliquots of the purified rHcp1 proteins were stored at -80°C.

### SDS-PAGE and Western blot

Protein samples were solubilized in 5 × SDS-PAGE sample buffer and heated to 100°C for 10 min. After centrifugation, the sample supernatants were loaded and proteins were separated on 12% SDS-PAGE. Proteins were visualized *via* staining with Coomassie blue R-250. For Western blot, proteins on the PAGE gel were electrotransferred onto a PVDF membrane (Beyotime). The membrane was blocked with 5% skim milk in high-salt Tris-buffered saline (HS-TBS; 20 mM Tris, 500 mM NaCl, pH 7.5) for 60 min at room temperature. Then, the membrane was incubated overnight at 4°C with a 1:10,000 dilution of the *B. pseudomallei* Hcp1-specific polyclonal antibody. After washed five times with PBS-T (PBS containing 0.05% Tween-20), the membrane was incubated for 1.0 h at 37°C with 1:5,000 of goat-anti-mouse IgG-horseradish peroxidase conjugate (Solarbio). Blot was visualized using the Pierce ECL Western blotting substrate (Thermo Scientific) and a ChemiDoc XRS imaging system (Bio-Rad).

### Median lethal dose (LD50) determination

Female BALB/c mice (aged 6–8 weeks) were purchased from the Animal Center of Army Medical University (Third Military Medical University) and acclimated in an ABLS-2^+^ laboratory for five days before experiments. All mice were fed an autoclaved pellet diet and sterile water under the following housing conditions: 23°, 50% humidity, half-day light/dark periods, and quiet surroundings with minimal disturbance. The experimental procedures for animals were performed according to the Regulations for Administration of Affairs Concerning Experimental Animals approved by Chinese State Council. Cervical dislocation was applied as euthanasia way of the experimental animals.

For LD50 determination, 50 female mice were challenged through intraperitoneal route with different concentrations of *B. pseudomallei* strain BPC006 (3.0 × 10^5^, 6.0 × 10^5^, 1.0 × 10^6^, 3.0 × 10^6^, and 6.0 × 10^6^ CFU, *n* = 10 per group). Infected mice were monitored for survival over 21 days, and the LD50 was calculated using Bliss method by SPSS software.

### Mouse vaccination and infection

Anesthetized BALB/c mice were grouped and administered in multisite routes (i.e., subcutaneous, intramuscular, and intraperitoneal) by three-dose vaccination regimens with (i) 100 µL PBS, (ii) 50 µg ^Δagr^MVs (in 100 µL PBS) prepared from *S. aureus* RN4220-Δ*agr* (RN), (iii) 50 µg ^hcp1^MVs derived from *S. aureus* RN-Hcp1, (iv) 50 µg ^hcp1^MVs emulsified with 50% Freund’s adjuvant (Sigma, USA), and (v) 0.2 µg rHcp1 proteins (equal to the amount of Hcp1 in 50 µg ^hcp1^MVs) on days -30, -20 and -10 prechallenge. Ten days after the third boost (day 0), the vaccinated mice were infected *via* intraperitoneal with lethal dose (5 × LD50) of *B. pseudomallei* BPC006. Challenged mice were monitored for behavior changes and survival time over 21 days.

### Detection of *B. pseudomallei* specific antibodies

During the process of mouse vaccination, sera from immunized mice (*n* = 3) seven days after the last vaccination (day -3 prechallenge) were collected, and antibodies against *B. pseudomallei* Hcp1 were determined by enzyme-linked immunosorbent assay (ELISA) as described (30). Briefly, 96-well Maxisorp plates (Nunc) were coated overnight at 4°C with rHcp1 (50 ng/mL) solubilized in carbonate buffer (pH 9.6). The plates were blocked at room temperature for 1.0 h with StartingBlock T20 blocking buffer (Pierce). After blocking, plates were washed three times with TBS-T (Tris-buffered saline supplemented 10% StartingBlock T20 and 0.05% Tween 20, pH 7.5). Then, twofold dilutions of serum samples were made with TBS-T, added in triplicate to wells, and incubated for 2.0 h at 37°C. After washed three times with TBS-T, the plates were incubated for 1.0 h at 37°C with goat anti-mouse IgG-horseradish peroxidase conjugate (1:5,000 dilution, Solarbio). After incubation, the plates were washed three times with TBS-T and developed by the EL-ABTS Chromogenic Reagent kit (Sangon Biotech) and read at 405 nm by using a FLUOstar Omega microplate reader (BMG Labtech). The reciprocals of the highest dilutions exhibiting optical densities (OD) that were 2.1 times relative to the normal mouse serum levels were used to determine the endpoint titers of antibodies for individual vaccinated mice.

### Detection of inflammatory factors

Serum samples were collected from individual vaccinated mice at 6 h after prime vaccination (day -30), days -20, -10, and -3 prechallenge, and days 0.5, 1, 2, and 5 postchallenge (*n* = 3 per time point). Then, the levels of inflammatory factors TNF-α and IL-6 in each serum samples were determined with an ELISA kit according to the manufacturer’s instructions (R&D Systems, USA). Results were examined with an ELISA reader Multiskan Mk3 (Thermo Fisher Scientific) at 450 nm, and inflammatory factor levels were determined according to the standard curve.

### Bacterial burden in organs of infected mice

The organs, including lungs, livers, and spleens, were collected from vaccinated mice on day 0.5 and 5 postchallenge, and subjected to colony forming unit (CFU) enumeration with a plate dilution method. Briefly, vaccinated mice (*n* = 50 for PBS group, *n* = 25 per other groups) were infected *via* intraperitoneal with lethal dose (5 × LD50) of *B. pseudomallei* BPC006 on day 0. Twelve infected mice on day 0.5 were sacrificed for organ bacterial counting (*n* = 10) and histological evaluation (*n* = 2). The remained mice were sacrificed on day 5 postchallenge. For bacterial counting, mouse organs were harvested, weighed, and homogenized in PBS using Covidien Precision tissue grinders (Fisher Scientific). Tissue homogenates were tenfold diluted in PBS, plated on LB agar, and incubated for 48 h at 37°C. Colonies were counted.

### Histological evaluation of mouse organs

The lungs, livers, and spleens collected from vaccinated mice (*n* = 2) on day 0.5 and 5 postchallenge were used for histological evaluation. Individual organ was fixed with 10% normal buffered formalin. The fixed tissues were embedded in paraffin and sectioned prior to staining with hematoxylin-eosin (HE). Tissue sections were observed under a microscope (Olympus BX53, Japan) and photographed.

### Statistical analysis

All experiments were conducted at least three times. Statistical analysis was performed using GraphPad Prism 8.0 software. The one-way analysis of variance (ANOVA), or two-way ANOVA method was used to compare the means from multiple groups and assess the statistical significance. All values were expressed as the mean ± standard derivations (SD) or standard error of the means (SEM). Significant difference was expressed as *P < 0.05, **P < 0.01, or ***P < 0.001.

## Results

### Construction of *S. aureus* strain producing Hcp1-loaded membrane vesicles

The *hcp1* gene of *B. pseudomallei* strain BPC006 was amplified with PCR and genetically fused to the 3′-terminus of the *pdhB* gene in *S. aureus* mutant RN4220-Δ*agr* (RN) to load Hcp1 into staphylococcal MVs. The in-frame fusion of target genes was verified by PCR amplification and DNA sequencing (**Supplementary Figure 1**), and the resultant strain was designated as RN4220-Δ*agr*/*pdhB-hcp1* (RN-Hcp1).

**Figure 1:**
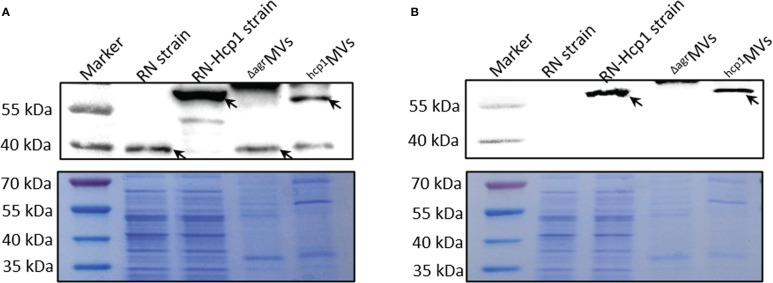
Characterization of PdhB-Hcp1 fusion proteins in *S. aureus* RN-Hcp1 strain and its MVs. Western blot with anti-PdhB **(A)** and anti-Hcp1 **(B)** antibodies, respectively. The top panels showed the blots of target proteins (indicated by black arrows) in bacterial cell lysates of *S. aureus* RN and RN-Hcp1 strains and their MVs, including ^Δagr^MVs and ^hcp1^MVs. The proteins in samples of interest were separated by 12% SDS-PAGE, stained with Coomassie blue R-250 and served as loading controls (bottom panels). The sizes of the protein Marker were indicated on the left.

The MVs produced by engineered *S. aureus* RN-Hcp1 (^hcp1^MVs) were prepared, and Western blot revealed the existence of a successful fusion protein with molecular weight of approximately 57 kDa in the total cell lysates of RN-Hcp1 and ^hcp1^MVs by using PdhB polyclonal antibodies as probe. *S. aureus* mutant RN and its derived ^Δagr^MVs presented wild-type PdhB with a low molecular weight (37 kDa, **Figure 1A** and **Supplementary Figure 2**). When polyclonal antibodies against Hcp1 were used, the protein of interest was only detected in the total cell lysates of RN-Hcp1 and ^hcp1^MVs but not in the RN mutant and ^Δagr^MVs (**Figure 1B**). TEM revealed the varied sizes of the purified MVs (**Supplementary Figure 3A**), and immuno-electron microscopy showed the membrane location of Hcp1 proteins (**Supplementary Figures 3B, C**). These data indicated that the engineered *S. aureus* RN-Hcp1 could successfully produce Hcp1-loaded MVs.

### General manifestation and humoral immune response in mice after Hcp1-loaded MV immunization

**Figure 2:**
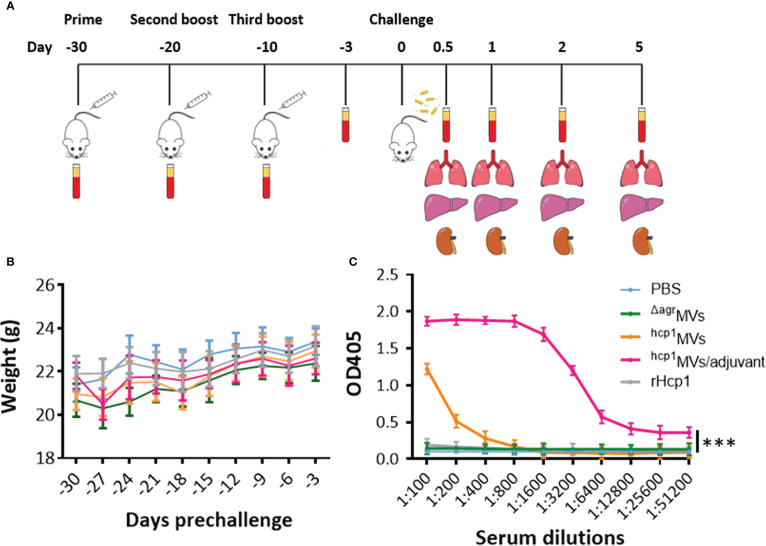
Body weight dynamics and serum antibody response of the vaccinated mice. **(A)** Experimental design and timeline of the regimen of PBS, ^Δagr^MVs, ^hcp1^MVs, ^hcp1^MVs/adjuvant, and rHcp1 vaccination, bacterial challenge, and sample collection. **(B)** Body weight dynamics of the vaccinated mice. Immunized BALB/c mice (*n* = 10 per group) were weighed every three days, and the weight of all groups increased gradually during vaccination. **(C)** Quantitation of serum antibody titers raised against rHcp1. The serum IgG antibody titers of immunized mice (*n* = 3 per group) on day -3 prechallenge were determined by ELISA and defined as the highest serum dilution that exhibited an optical density (OD) value of 2.1 times relative to the negative control (PBS group serum). Statistical significance was calculated by two-way ANOVA, ***P < 0.001.

BALB/c mice were immunized with PBS, ^Δagr^MVs, ^hcp1^MVs, ^hcp1^MVs/adjuvant, and recombinant Hcp1 (rHcp1, *n* = 10 per group) by a three-dose regimen at 10-day intervals *via* multiple routes (i.e., subcutaneous, intramuscular, and intraperitoneal) to assess the effects of Hcp1-loaded MVs on tested animals (**Figure 2A**). The body weights of MVs- or rHcp1-challenged mice showed no difference during the immunization process compared with those of the PBS-challenged mice (**Figure 2B**). All vaccinated mice exhibited good mental state, smooth fur, normal food and water intake, normal posture and gait, and comparable nutrition. Sera from vaccinated mice (*n* = 3 per group) on day -3 prechallenge were used to determine antibody responses to rHcp1 by using indirect ELISA. Results revealed that the titer of the total serum IgG antibodies from ^hcp1^MVs/adjuvant-immunized mice was highest among all groups (**Figure 2C**). The sera from mice that received a prime and two boosts of ^hcp1^MVs vaccination demonstrated higher IgG titers than those from rHcp1- and ^Δagr^MVs-vaccinated mice (**Figure 2C**), suggesting a partial adjuvant effect of staphylococcal MVs.

### Inflammatory factor production following Hcp1-loaded MV immunization

**Figure 3:**
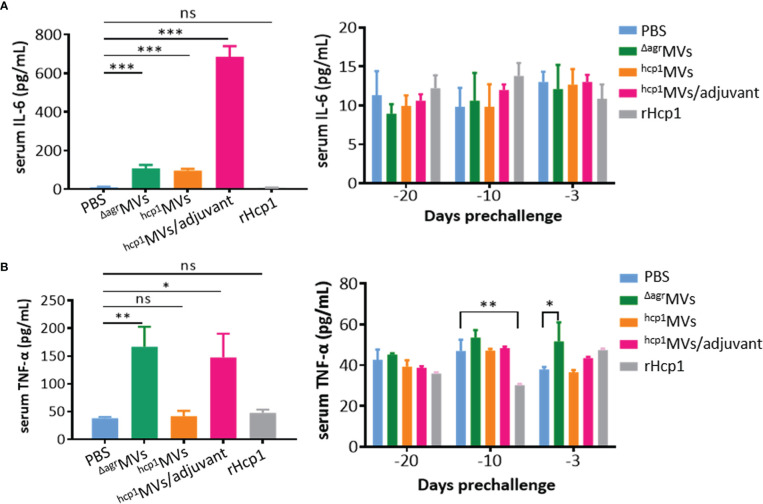
Inflammatory factor production determined by ELISA. BALB/c mice (*n* = 3 per group) were each vaccinated with PBS, ^Δagr^MVs, ^hcp1^MVs, ^hcp1^MVs/adjuvant, or rHcp1 on days -30, -20, and -10. Serum samples were collected at 6 h after prime vaccination (day -30, left panels) and days -20, -10 and -3 preinfection (right panels). ELISA was performed to quantitate serum IL-6 **(A)** and TNF-ɑ **(B)** levels with commercial kits (R&D Systems). The assay was repeated in triplicate. Statistical significances were calculated by one-way ANOVA; ns indicates no statistical significance, *P < 0.05, **P < 0.01, and ***P < 0.001.

The levels of inflammatory factor production present the systemic inflammation of body caused by bacteria or toxins (31). However, certain antigens or adjuvants can activate immune cells to secrete cytokines, such as TNF-α, IL-6, and IL-12, that are involved in the promotion of Th1 immune responses (32). Sera from BALB/c mice vaccinated with PBS, ^Δagr^MVs, ^hcp1^MVs, ^hcp1^MVs/adjuvant, and rHcp1 (*n* = 3 per time point) were collected at 6 h after prime vaccination (day -30) and days -20, -10, and -3 preinfection, and subjected to quantification of IL-6 and TNF-α levels by using ELISA. As shown in **Figure 3A**, the IL-6 levels in mice 6 h after prime vaccination (day -30) with ^Δagr^MVs, ^hcp1^MVs, and ^hcp1^MVs/adjuvant were significantly increased compared with those in PBS- and rHcp1-vaccinated mice (*P* < 0.001). The TNF-α level in immunized mice 6 h after prime vaccination of ^hcp1^MVs/adjuvant and ^Δagr^MVs remarkably increased relative to that in PBS-challenged mice, whereas comparable TNF-α level was observed in mice 6 h after prime vaccination of ^hcp1^MVs and rHcp1. Furthermore, the IL-6 and TNF-α levels of ^Δagr^MVs-, ^hcp1^MVs-, ^hcp1^MVs/adjuvant-, and rHcp1-injected group on days -20, -10, and -3 were low and comparable with those of PBS-injected group (**Figure 3B**). These data indicated that the Hcp1-loaded MV vaccine was safe and only elicited increased inflammatory factor production during early stage of prime immunization.

### Protective capacity of Hcp1-loaded MV vaccine against *B. pseudomallei* infection

**Figure 4:**
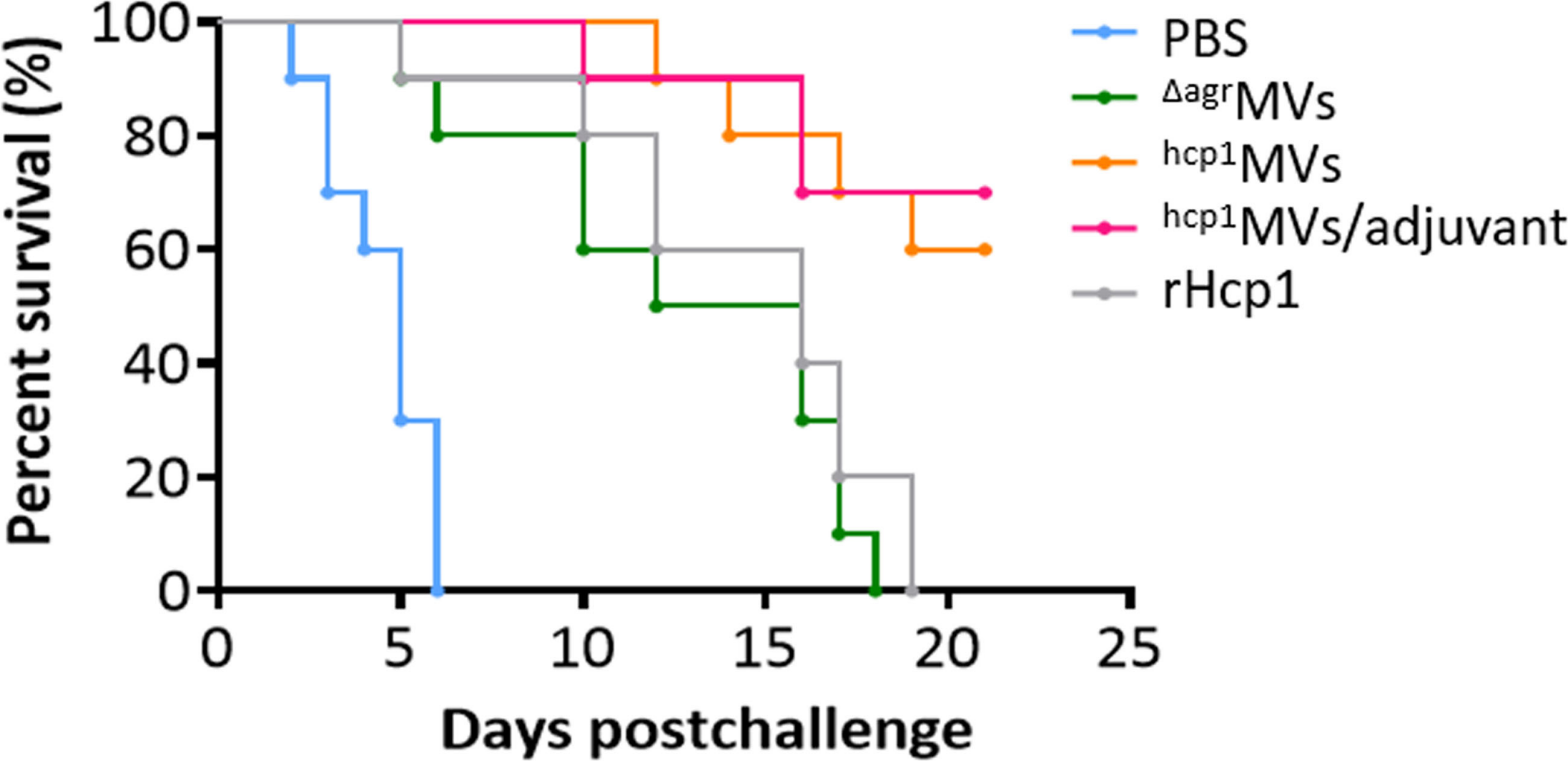
Hcp1-loaded MV vaccination protects mice from acute melioidosis. BALB/c mice (*n* = 10 per group) were vaccinated with PBS, ^Δagr^MVs, ^hcp1^MVs, ^hcp1^MVs/adjuvant, or rHcp1 by a three-dose regimen at 10-day intervals. Then, mice were infected with 5 × LD50 of *B. pseudomallei* BPC006 (lethal dose) 10 days after the final boost. Mouse mortality was evaluated daily for 21 days and the percent survival (%) was indicated.

The median lethal dose (LD50) was determined to evaluate the protective repertoire of Hcp1-loaded MV against *B. pseudomallei* challenge. Female BALB/c mice were challenged intraperitoneally with various dosages of *B. pseudomallei* BPC006 (3.0 × 10^5^, 6.0 × 10^5^, 1.0 × 10^6^, 3.0 × 10^6^, and 6.0 × 10^6^ CFU). Mouse survival was monitored for 21 days postinfection, and the Bliss method of SPSS software showed that the LD50 was 5.75 × 10^5^ CFU (**Supplementary Figure 4**). BALB/c mice were vaccinated with PBS, ^Δagr^MVs, ^hcp1^MVs, ^hcp1^MVs/adjuvant, or rHcp1 (*n* = 10 per group) by a three-dose regimen at 10-day intervals and then infected with lethal dose of *B. pseudomallei* BPC006 (5 × LD50) 10 days after the final boost to evaluate the protective capacity of the MV vaccine. All infected mice in the PBS-treated group succumbed rapidly to the lethal challenge of *B. pseudomallei* BPC006 (< 7 days). ^Δagr^MVs- or rHcp1-vaccinated mice survived less than 20 days after *B. pseudomallei* infection. By contrast, immunization with ^hcp1^MVs and ^hcp1^MVs/adjuvant protected 60% and 70% of mice, respectively, from lethal bacterial challenge with surviving time exceeding 21 days (**Figure 4**).

### Inflammatory cytokine production in vaccinated mice after *B. pseudomallei* infection

**Figure 5:**
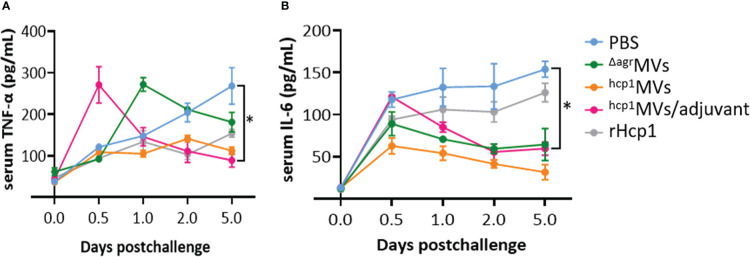
The inflammatory cytokine levels in sera of vaccinated mice after *B. pseudomallei* challenge. Mice (*n* = 3 per group) were vaccinated with PBS, ^Δagr^MVs, ^hcp1^MVs, ^hcp1^MVs/adjuvant, or rHcp1 by three inoculations at 10-day intervals. Ten days after the final boost, mice were infected with lethal dose of *B. pseudomallei* BPC006 (5 × LD50) *via* intraperitoneal injection. Serum samples were harvested on days 0.5, 1, 2, and 5 postinfection and the levels of inflammatory cytokines, including TNF-α **(A)** and IL-6 **(B)**, were determined by ELISA. Data are presented as mean ± SD. Statistical significance was calculated by two-way ANOVA, *P < 0.05.

Serum samples of vaccinated mice on days 0.5, 1, 2, and 5 postinfection of *B. pseudomallei* BPC006 were collected and subjected to the determination of inflammatory cytokine levels. As shown in **Figure 5**, the levels of TNF-α and IL-6 in PBS-treated mice five days after lethal bacterial exposure were significantly elevated than those in other groups and increased over time. By contrast, the TNF-α and IL-6 levels in ^hcp1^MVs- and ^hcp1^MVs/adjuvant-immunized mice five days postchallenge decreased continuously after day 0.5 of infection (except TNF-α). Notably, inflammatory cytokine levels in the ^hcp1^MVs/adjuvant-vaccinated mice increased remarkably after day 0.5 of exposure, decreased rapidly, and then maintained at a low level. The increased inflammatory cytokine levels during early period after *B. pseudomallei* exposure may contribute to bacterial clearance (32).

### Bacterial burden in vaccinated mice after bacterial challenge

**Figure 6:**
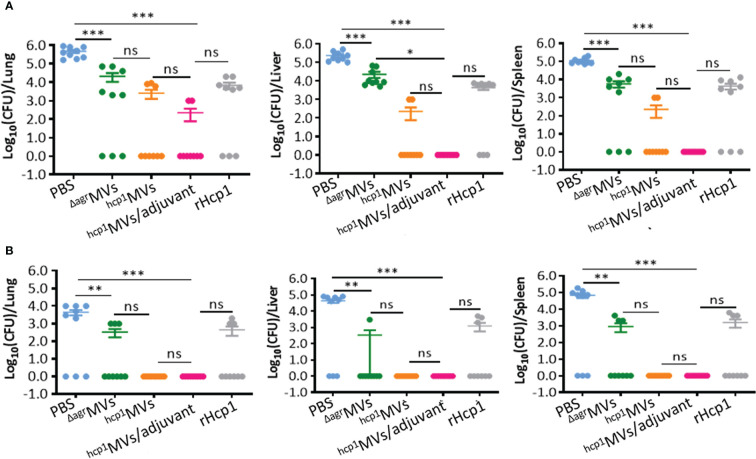
Bacterial enumeration in vaccinated mice challenged with *B. pseudomallei*. Viable bacteria in lungs, livers, and spleens of immunized mice (*n* = 9 per group) on day 0.5 **(A)** and 5 **(B)** postinfection. Tissues were weighed and homogenized in PBS. Viable bacteria were counted using the plate dilution method and calculated as CFU. Data are presented as mean ± standard error of the mean (SEM). Significant differences were analyzed by one-way ANOVA, ns indicates no difference, *P < 0.05, **P < 0.01, and ***P < 0.001.

Powerful vaccines resulted in bacterial clearance *in vivo*. BALB/c mice were vaccinated with PBS, ^Δagr^MVs, ^hcp1^MVs, ^hcp1^MVs/adjuvant, or rHcp1 (*n* = 9 per group), and tissue samples of lungs, livers, and spleens were obtained from immunized mice on day 0.5 and 5 postchallenge with lethal *B. pseudomallei*. Bacterial enumeration revealed that the viable bacteria in collected organs of the ^hcp1^MVs- and ^hcp1^MVs/adjuvant-immunized mice on day 0.5 postchallenge were decreased than those of other groups (**Figure 6A**). Moreover, ^hcp1^MVs- and ^hcp1^MVs/adjuvant- immunized mice demonstrated undetectable viable bacteria in the three tissues on day 5 postinfection (**Figure 6B**). Results also showed that all mice immunized with ^hcp1^MVs or ^hcp1^MVs/adjuvant exhibited no detectable bacteria in their tissues on day 5 postinfection (**Figure 6B**). These data indicated that Hcp1-loaded MV vaccine is a promising candidate for *B. pseudomallei* clearance and protects against acute melioidosis.

### Histopathological analysis of infected mouse tissues

The lungs, livers, and spleens of mice immunized with PBS, ^Δagr^MVs, ^hcp1^MVs, ^hcp1^MVs/adjuvant, or rHcp1 (*n* = 2 per group) were collected on day 0.5, 1, 2, and 5 postchallenge with *B. pseudomallei* BPC006 and subjected to histopathological analysis. In the gross view, the normal morphologies of livers, spleens, and lungs were maintained in ^hcp1^MVs/adjuvant-vaccinated mice after five days of infection. However, small white abscesses formed in the livers, spleens, and lungs of PBS-immunized mice five days postinfection and enlarged spleens and lungs with mucinous exudate on the surface were also observed (**Supplementary Figure 5**).

**Figure 7:**
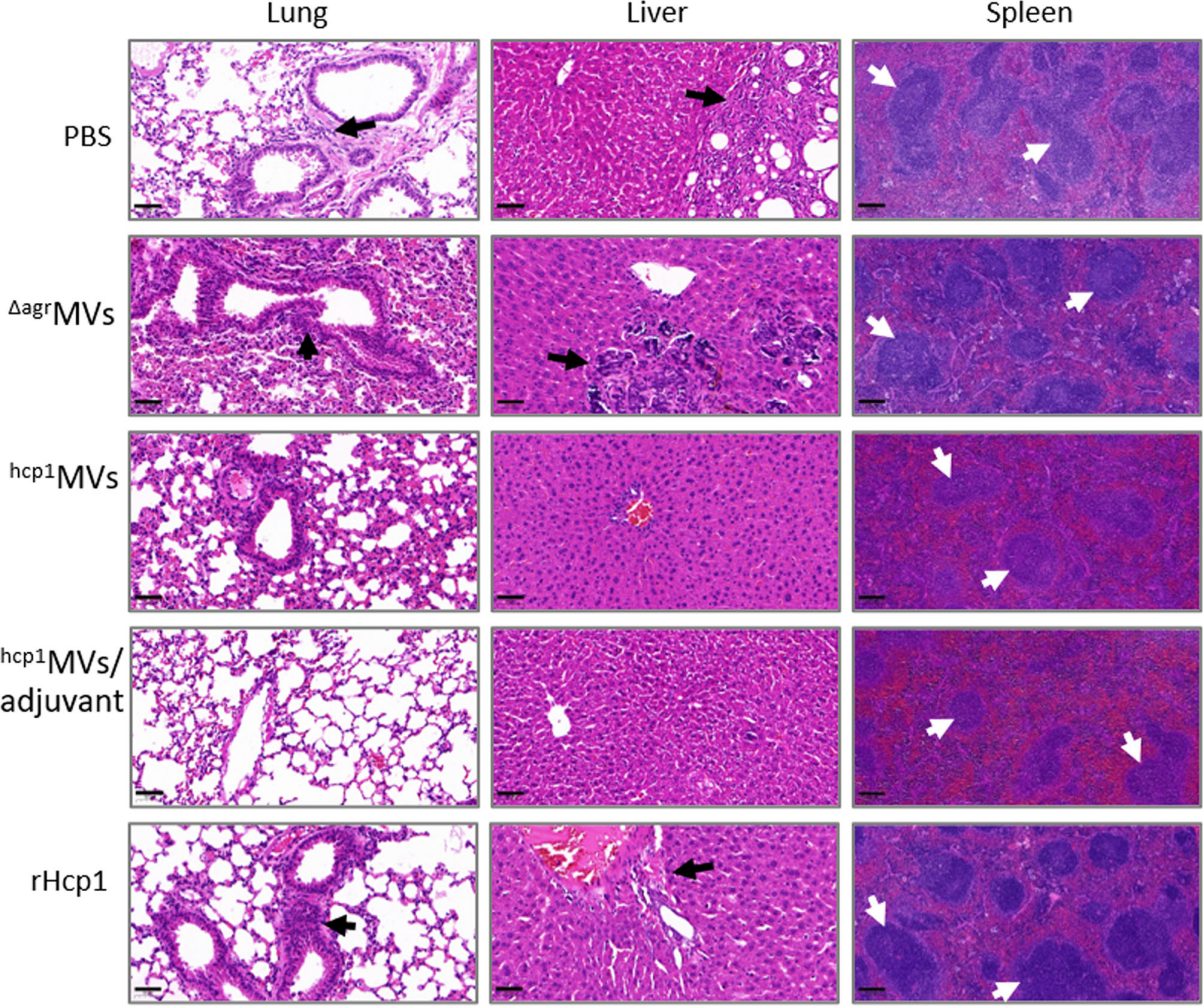
Reduced organ inflammations in vaccinated mice after exposure to *B. pseudomallei*. The lungs, livers, and spleens (*n* = 2 per group) of vaccinated mice on day 5 postchallenge were collected and fixed in 10% formalin. Then, tissues were paraffin-embedded, sectioned, stained with hematoxylin and eosin, and subjected to pathological examination. The representative field for certain tissues was observed at 20× (bar = 50 µm, lung and liver) and 10× (bar = 200 µm, spleen) magnifications under a microscope. White arrows indicated multinucleated giant cells, whereas black arrows showed inflammatory infiltrations.

Tissue sections, including lungs, livers, and spleens, were prepared, stained with hematoxylin and eosin (HE), and examined in a blind manner by a pathologist. Histopathological analysis revealed that the lungs, livers, and spleens from ^hcp1^MVs/adjuvant-immunized mice on day 5 postinfection exhibited normal tissue architecture (**Figure 7**). By contrast, the lungs, livers, and spleens of animals vaccinated with ^hcp1^MVs were almost normal except for some areas of mild interstitial inflammation. The bronchovascular inflammatory infiltrations were observed in lungs of ^Δagr^MVs- or rHcp1-vaccinated mice on the indicated days postchallenge (**Supplementary Figure 6**). Bronchovascular infiltration was a prevalent characteristic in the lungs of mice vaccinated with PBS. In addition, frequent interstitial inflammation and multinucleated giant cell infiltration were also observed in the spleens of PBS-vaccinated mice five days after bacterial challenge (**Figure 7**). Overall, these data demonstrated the vaccinogenic potential of Hcp1-loaded staphylococcal MVs.

## Discussion

Despite the fact that licensed vaccines are unavailable, many vaccine candidates against melioidosis, including inactivated whole-cell (32, 33), live attenuated (13, 34), subunit (14, 15), glycoconjugate (35, 36), DNA (37, 38), and viral vector-based (39) vaccines, have been studied in recent years. However, these vaccines only have partial protection probably because *B. pseudomallei* is an intracellular organism that survives in macrophages, neutrophils, and monocytes, thus avoiding the induction of protective immune responses (40–43). Nieves et al. prepared MVs naturally derived from *B. pseudomallei* strain 1026b (44) and showed that the vaccination of BALB/c mice with MVs provides considerable protection against septic (100%) and pneumonic (60%) melioidosis (44, 45). However, natural MVs are commonly enriched with virulence factors, such as lipopolysaccharide (21, 25), and the application of MVs produced by wild-type *B. pseudomallei* strains raises safety concerns. In a previous study, a whole gene locus deletion of *agr* system is used to generate an *S. aureus* strain RN4220-Δ*agr* (25). MVs prepared from RN4220-Δagr (^Δagr^MVs) are remarkably attenuated, and PdhB is the most abundant component among ^Δagr^MVs that is suitable for the delivery of heterogeneous antigens from bacteria to the secreted MVs. On the basis of this safe protein delivery platform, the gene of *B. pseudomallei* protective antigen Hcp1 is genetically fused with *pdhB* in *S. aureus* RN4220-Δ*agr*, and the protective immune responses generated by ^hcp1^MVs vaccination against acute melioidosis are evaluated in the present study.

Recent studies showed that antibody responses play a critical role in the protection of live attenuated vaccine-vaccinated mice from *B. pseudomallei* infection and that bacterium-specific cellular responses make a minor contribution (34, 46). In this study, vaccination with rHcp1 proteins without adjuvants in a three-dose regimen has produced indistinguishable level of antibodies compared with the PBS control (**Figure 2C**), suggesting the requirement of immune adjuvants, such as Freund’s adjuvant (47), Sigma adjuvant system (19), and CpG DNA (37), in subunit vaccine immunization. By contrast, the vaccination of mice with Hcp1-loaded MVs (^hcp1^MVs) in the absence of adjuvants produces considerable humoral responses (serum IgG titer > 1:400) compared with the vaccination of mice with ^Δagr^MVs that do not encapsulate Hcp1 (**Figure 2C**), indicating an adjuvant effect of staphylococcal ^Δagr^MVs for Hcp1. However, the adjuvant effect of ^Δagr^MVs remains partial because the immunization of mice with a formulation of ^hcp1^MVs/adjuvant induces high specific antibodies (IgG titer > 1:51,200, **Figure 2C**). The self-adjuvant activity of MVs is probably due to the presence of vesicle-related pathogen-associated molecular patterns (PAMPs), which can bind pathogen recognition receptors on innate immune cells to enhance antigen-presenting functions (48). The MV adjuvant activity is remarkably correlated with the type and amount of PAMPs incorporated in vesicles (48, 49). As heterogeneous MVs for *B. pseudomallei* Hcp1, PAMPs and their functions in staphylococcal ^Δagr^MVs deserve further investigation.

As a subunit vaccine, rHcp1 is previously prepared and mixed with Sigma adjuvant, and BALB/c mice are vaccinated by three inoculations of 10 µg rHcp1 proteins at 2-week intervals and infected with *B. pseudomallei* K96243 strain three weeks after the final boost. Results showed that the rHcp1/adjuvant can protect 50% of mice from lethal dose challenge but fails to prevent chronic colonization (19). In this study, the inability to prevent acute melioidosis is observed after vaccination of BALB/c mice with ^Δagr^MVs or rHcp1 protein only (**Figure 4**). Notably, a similar post-challenge survival pattern was observed between rHcp1- and ^Δagr^MVs- immunized mice. Vaccination of rHcp1 without adjuvant formulation can enhance the post-challenge survival of BALB/c mice against *B. pseudomallei. S. aureus* derived ^Δagr^MVs do not have specific antigens of *B. pseudomallei*, however, ^Δagr^MVs can exhibit intrinsic adjuvant activity (25). The PAMPs involved in ^Δagr^MVs may activate innate immune cells, such as dendritic cells and monocytes/macrophages, to provide certain roles in pathogen inactivation (48). By contrast, engineered ^hcp1^MVs protects 60% of BALB/c mice against lethal challenge with BPC006, and the formulation of ^hcp1^MVs/adjuvant protects 70% of mice against acute melioidosis. Compared with the rHcp1 protein/adjuvant formulation (19), the elevated protective ability of ^hcp1^MVs may be ascribed to the intrinsic adjuvanticity of staphylococcal MVs. PAMPs encapsulated in bacterial MVs can also activate immune cells to secrete cytokines and promote a Th1 immune response (32). Consistently, the serum levels of TNF-α and IL-6 in mice 6 h after prime vaccination (day -30) with ^hcp1^MVs/adjuvant are significantly increased compared with those in the PBS control group. The serum TNF-α and IL-6 levels in mice vaccinated with ^Δagr^MVs are also remarkably increased and then gradually decreased to normal levels on days -20, -10, and -3 prechallenge (**Figure 3**). This result is consistent with the finding in the previous study that ^Δagr^MVs inoculation induces lower level of inflammatory factor production than MVs derived from the wild-type *S. aureus* strain (25). However, the inflammatory cytokine levels, especially TNF-α, in the ^hcp1^MVs/adjuvant-vaccinated mice increased remarkably on day 0.5 after *B. pseudomallei* exposure (**Figure 5**). The increased inflammatory cytokine levels during early period after infection may contribute to bacterial clearance (32), which is partially confirmed by the bacterial counting from infected mouse organs (**Figure 6A**). By contrast, ^hcp1^MVs-vaccinated animals have low serum TNF-α and IL-6 levels throughout the experimental period. The reason behind this phenomenon is unclear, and the association between ^hcp1^MV-stimulated inflammatory factor level and its protective potential is of interest and worthy of further study.

A major challenge in developing vaccines against diseases caused by facultative intracellular pathogens is the ability of the host to achieve sterilizing immunity (50). Importantly, vaccination with ^hcp1^MVs or ^hcp1^MVs/adjuvant can achieve sterilizing immunity. Culturable bacteria are not determined in lungs, livers, and spleens of ^hcp1^MVs- or ^hcp1^MVs/adjuvant-vaccinated mice on day 5 postinfection of *B. pseudomallei* BPC006, whereas most PBS-inoculated mice (66.7%) have bacterial burden in their organs (**Figure 6B**). In addition, the organ tissue histopathology of vaccinated mice after challenge with *B. pseudomallei* was assessed, and data showed decreased histopathological changes, reduced interstitial inflammation foci, and declined multinucleated giant cell numbers in ^hcp1^MVs- and ^hcp1^MVs/adjuvant- vaccinated mice compared with those in PBS-injected mice (**Figure 7**). These discrete organ bacterial burdens and histopathological observations indicate that the vaccination of Hcp1-loaded MVs contributes to the induction of specific immune responses that control the infection and prevent disseminated melioidosis disease in various organs. Our observations correlate well with the previous finding that pathogen clearance is observed in *B. mallei* live attenuated vaccine-vaccinated mice after challenge with bacteria (88% clearance) (34).

In conclusion, our data demonstrate that Hcp1, a tube component and secreted effector of T6SS in *B. pseudomallei*, is an effective vaccine candidate and that engineered *S. aureus* RN4220-Δ*agr*/*pdhB-hcp1* can successfully produce ^hcp1^MVs that have protection potential against *B. pseudomallei* acute infections. We have reached an interesting partial adjuvant activity of staphylococcal MVs. The addition of an adjuvant to ^hcp1^MVs will further improve the protection ability and stimulate an enhanced sterilizing immunity for complete bacterial clearance. Overall, our results present a new way to develop melioidosis vaccines and provide valuable insights into the development of an antigenically defined, safe, and effective MV vaccine.

## Data availability statement

The original contributions presented in the study are included in the article/**Supplementary Material**. Further inquiries can be directed to the corresponding authors.

## Ethics statement

The animal study was reviewed and approved by Third Military Medical University.

## Author contributions

XR, RZ, and ML designed experiments, and revised manuscript. KZ, GL, MZ, XP, and JL performed experiments and interpreted data. KZ, GL, and YR investigated data and drew figures. XR and YR edited manuscript. All authors reviewed drafts and approved the submitted version.
