# Supplementary material for: Hcp1-loaded staphylococcal membrane vesicle vaccine protects against acute melioidosis
Source: Front Immunol. 2022 Dec 23;13:1089225. doi: 10.3389/fimmu.2022.1089225 (PMC9822774; doi:10.3389/fimmu.2022.1089225)
Supplement: Supplementary file 1 [file DataSheet_1.pdf]

Supplementary Materials

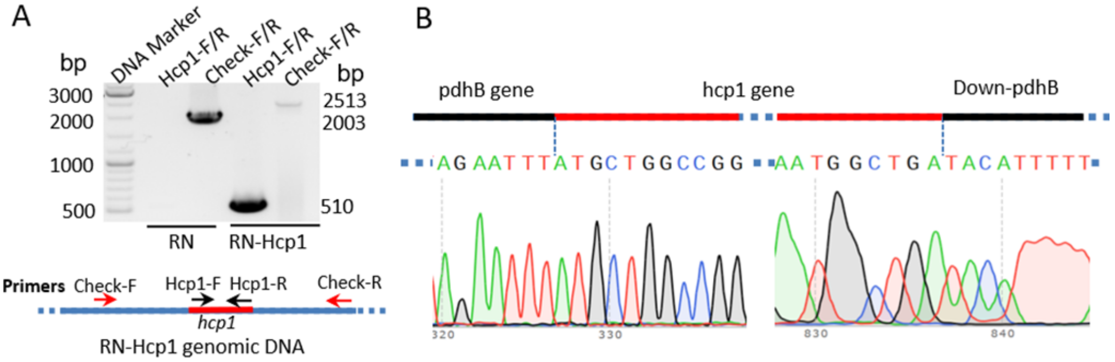

Supplementary Figure 1. Identification of *S. aureus* RN4220-Δ*agr*/*pdhB-hcp1* (RN-Hcp1). **(A)** Identification of RN-Hcp1 strain by PCR amplification. The primers used were shown in the bottom panel. The amplicon derived from RN-Hcp1 genomic DNA was larger than that from *S. aureus* RN, indicating the successful insertion of *hcp1* gene into the genomic DNA of RN-Hcp1 strain. The sizes of target fragments were indicated on the right. **(B)** Characterization of RN-Hcp1 by DNA sequencing. DNA sequencing of the PCR product further confirmed the successful in-frame fusion of *hcp1* gene to the *pdhB* in RN-Hcp1 strain.

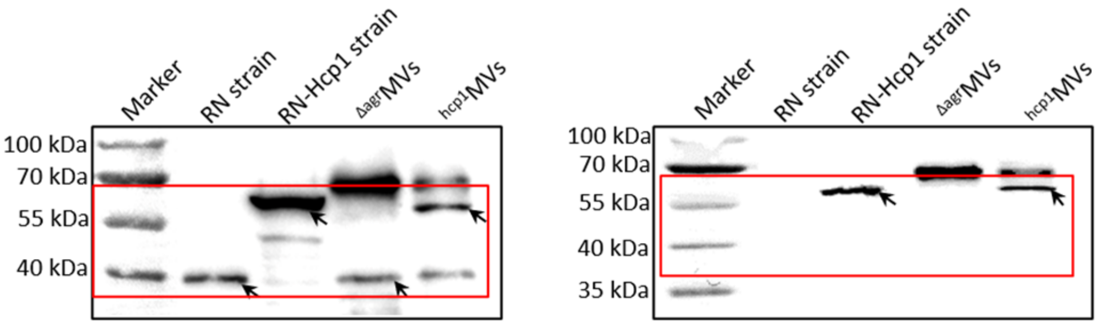

Supplementary Figure 2. Full Western blot data. The full-length blots for Western blot pictures were presented. The red boxes represented the depicted parts of the blot in Fig.1.

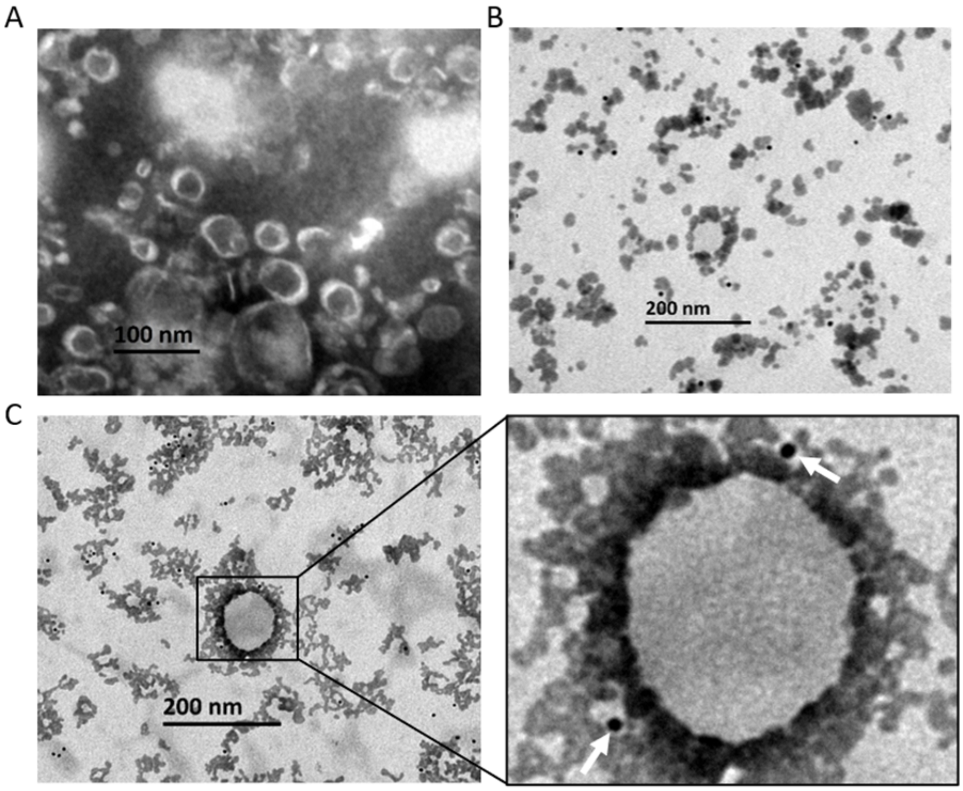

Supplementary Figure 3. Observation of the Hcp1-loaded MVs by transmission electron microscope (TEM). **(A)** Hcp1-loaded MVs produced by *S. aureus* strain RN-Hcp1 observed under TEM. The bar representing 100 nm was indicated. **(B)** Representative immunoelectron image of $^{\Delta agr}$MVs derived from *S. aureus* strain RN served as negative control. MV particles were mainly damaged. The bar representing 200 nm was indicated. **(C)** Representative immunoelectron micrograph of ^{hcp1}MVs contained Hcp1 detected. The image showed efficient labeling of Hcp1-fused proteins with mouse-anti-Hcp1 antibodies (indicated by white arrows).

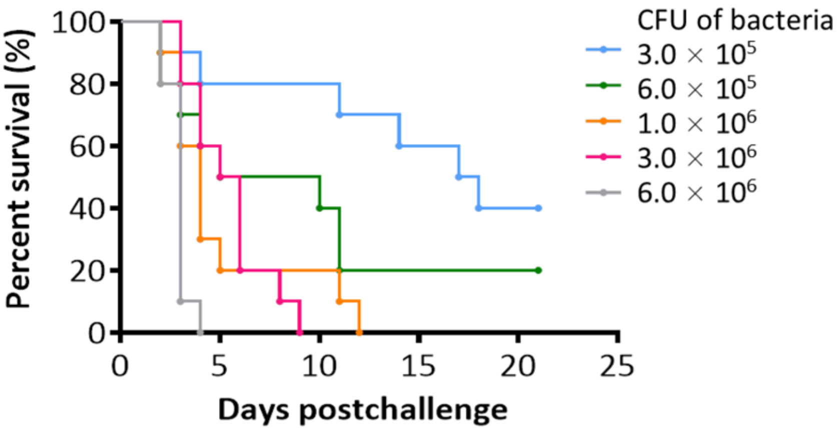

Supplementary Figure 4. LD50 determination for BALB/c mice against *B. pseudomallei* BPC006 infection. Female BALB/c mice (n = 10 per group) were exposed to 3.0×10^5, 6.0×10^5, 1.0×10^6, 3.0×10^6, or 6.0×10^6 CFU of *B. pseudomallei* strain BPC006 through intraperitoneal routes. Mouse survivals were monitored for 21 days. The LD50 was calculated by Bliss method of SPSS software.

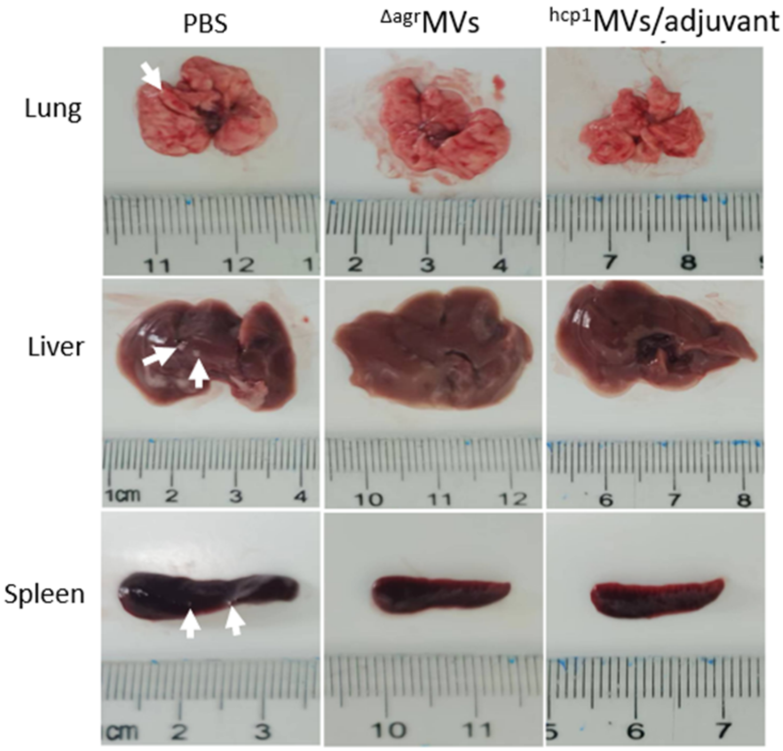

Supplementary Figure 5. Gross specimen of organs of the vaccinated mice five days after exposure to *B. pseudomallei* BPC006. The lungs, livers, and spleens of infected mice were collected and presented. Small white abscesses observed in the lung, liver, and spleen of PBS-immunized mice on day 5 postinfection were indicated by white arrows. The enlarged spleens and lungs of PBS-immunized mice with mucinous exudate on the surface were also observed.

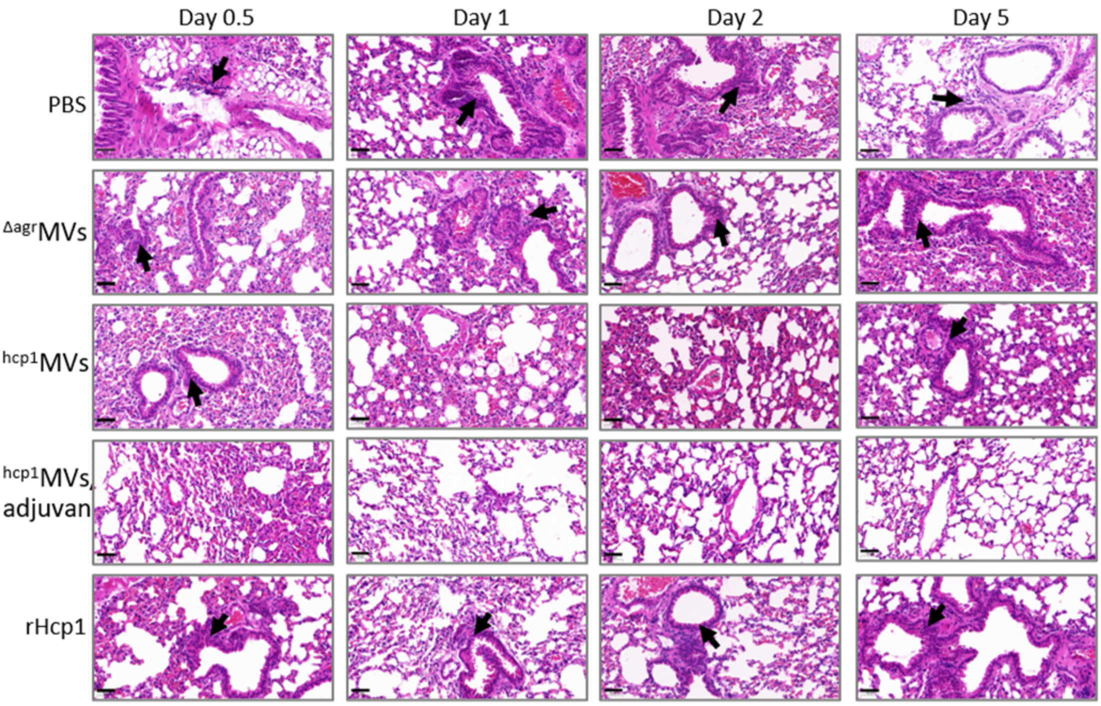

Supplementary Figure 6. Histological analysis revealed reduction of lung bronchovascular inflammatory infiltrations of ^{hcp1}MVs/adjuvant-vaccinated mice after exposure to *B. pseudomallei*. The lungs of vaccinated mice on days 0.5, 1, 2, and 5 after exposure to *B. pseudomallei* BPC006 were harvested and fixed in 10% formalin. Then, tissues were paraffin-embedded, sectioned, and stained with hematoxylin and eosin (HE). Representative views were taken at 20× magnification under a microscope. The scale bar represents 50 µm, and the bronchovascular inflammatory infiltrations were indicated by black arrows.

Supplementary Tables

Supplementary Table 1. Plasmids and bacterial strains used in this study

**Plasmids/strains**	**Reference/source**
**Plasmids**	
pBT2	(1)
pBT2-*hcp1*	This work
pET28a	Thermo Fisher Scientific
pET28a-*hcp1*	This work
**Strains**	
S. aureus RN4220	(2)
S. aureus RN4220-Δ*agr*	(3)
S. aureus RN4220-Δ*agr/pdhB-hcp1*	This work
B. pseudomallei BPC006	(4)
E. coli DH5α	Tiangen
E. coli BL21 (DE3)	Tiangen
E. coli BL21(DE3)/pET28a-*hcp1*	This work

Supplementary Table 2. Primers used for PCR amplification and cloning

Primers	Sequences (5'– 3')	Descriptions	Product sizes
uppdhB-F	agtgcagcggaattcgagctATGGCACAAATGACAA	For amplification of the left region before stop codon of the *pdhB* gene (Up-pdhB)	975 bp
uppdhB-R	cggccagcatAAATTCTAAAGTTTCTTTTGCTTTT		
downpdhB-F	gaatggctgaTACATTTTAAAAGTTAACGAAGTTAG	For amplification of the right region after stop codon of the *pdhB* gene (Down-pdhB)	967 bp
downpdhB-R	cctgcaggtcgactctagagTCCAGTAATGTTTATGAACG		
hcp1-F	tttagaatttATGCTGGCCGGAATATATCTC	For amplification of *hcp1* gene	510 bp
hcp1-R	ttaaaatgtaTCAGCCATTCGTCCAGTTG		
check-F	ATGGCACAAATGACAATGGTTCAA	For verification of gene *hcp1* insertion	2507 bp
check-R	ACTAATAATCCTCTATCAGTGTCTG		
pET28a-F	ggtgctcgagtgcggccgcaATGCTGGCCGGAATATATCTC	For construction of the Hcp1 expression plasmid	550 bp
pET28a-R	tggacagcaaatgggtcgcgTCAGCCATTCGTCCAGTTTG		

References

1. Bruckner R. Gene replacement in *Staphylococcus carnosus* and *Staphylococcus xylosus*. *FEMS Microbiol Lett* (1997) 151(1): 1–8. doi: 10.1111/j.1574-6968.1997.tb10387.x
2. Berscheid A, Sass P, Weber-Lassalle K, Cheung AL, Bierbaum G. Revisiting the genomes of the *Staphylococcus aureus* strains NCTC 8325 and RN4220. *Int J Med Microbiol* (2012) 302(2): 84–7. doi: 10.1016/j.ijmm.2012.01.002.
3. Yuan J, Yang J, Hu Z, Yang Y, Shang W, Hu Q, Safe staphylococcal platform for the development of multivalent nanoscale vesicles against viral infections. *Nano lett* (2018) 18(2): 725–33. doi: 10.1021/acs.nanolett.7b03893
4. Fang Y, Huang Y, Li Q, Chen H, Yao Z, Pan J, et al. First genome sequence of a *Burkholderia pseudomallei* isolate in China, strain BPC006, obtained from a melioidosis patient in Hainan. *J Bacteriol* (2012) 194(23): 6604–5. doi: 10.1128/JB.01577-12
